# Experimental validation of the Eclipse AAA algorithm

**DOI:** 10.1120/jacmp.v8i2.2350

**Published:** 2007-05-10

**Authors:** Karen Breitman, Satyapal Rathee, Chris Newcomb, Brad Murray, Don Robinson, Colin Field, Heather Warkentin, Sherry Connors, Marc MacKenzie, Peter Dunscombe, Gino Fallone

**Affiliations:** ^1^ Department of Medical Physics, Tom Baker Cancer Centre University of Calgary Calgary; ^2^ Departments of Oncology University of Calgary Calgary; ^3^ Departments of Radiology University of Calgary Calgary; ^4^ Department of Medical Physics, Cross Cancer Institute University of Alberta Edmonton Alberta Canada; ^5^ Department of Oncology University of Alberta Edmonton Alberta Canada

**Keywords:** AAA, TG‐53 criteria, dosimetric evaluation, photon‐beam dose calculation

## Abstract

The present study evaluates the performance of a newly released photon‐beam dose calculation algorithm that is incorporated into an established treatment planning system (TPS). We compared the analytical anisotropic algorithm (AAA) factory‐commissioned with “golden beam data” for Varian linear accelerators with measurements performed at two institutions using 6‐MV and 15‐MV beams. The TG‐53 evaluation regions and criteria were used to evaluate profiles measured in a water phantom for a wide variety of clinically relevant beam geometries. The total scatter factor (TSF) for each of these geometries was also measured and compared against the results from the AAA.

At one institute, TLD measurements were performed at several points in the neck and thoracic regions of a Rando phantom; at the other institution, ion chamber measurements were performed in a CIRS inhomogeneous phantom. The phantoms were both imaged using computed tomography (CT), and the dose was calculated using the AAA at corresponding detector locations. Evaluation of measured relative dose profiles revealed that 97%, 99%, 97%, and 100% of points at one institute and 96%, 88%, 89%, and 100% of points at the other institution passed TG‐53 evaluation criteria in the outer beam, penumbra, inner beam, and buildup regions respectively. Poorer results in the inner beam regions at one institute are attributed to the mismatch of the measured profiles at shallow depths with the “golden beam data.”

For validation of monitor unit (MU) calculations, the mean difference between measured and calculated TSFs was less than 0.5%; test cases involving physical wedges had, in general, differences of more than 1%. The mean difference between point measurements performed in inhomogeneous phantoms and Eclipse was 2.1% (5.3% maximum) and all differences were within TG‐53 guidelines of 7%. By intent, the methods and evaluation techniques were similar to those in a previous investigation involving another convolution–superposition photon‐beam dose calculation algorithm in another TPS, so that the current work permitted an independent comparison between the two algorithms for which results have been provided.

PACS number: 87.53.Dq

## I. INTRODUCTION

Accurately modeling the distribution of dose in clinical situations is essential to the modern practice of radiotherapy. Convolution–superposition algorithms have proved to be reasonably successful at modeling dose distributions over a wide range of conditions of varying complexity, including inhomogeneous media.^(^ ^1^ ^–^ ^3^ ^)^ The analytical anisotropic algorithm (AAA)^(^ ^4^ ^,^ ^5^ ^)^ is a new convolution–superposition‐based photon‐beam dose computation algorithm released in 2005 for use in an established commercial TPS (Eclipse: Varian Medical Systems, Palo Alto, CA).

The introduction of a new dose calculation algorithm into a commercial TPS warrants extensive validation by the medical physics community before the algorithm is accepted for clinical implementation. Thus there is an impetus to critically examine the performance of the AAA. Selecting a proper set of validation tests to be applied and identifying appropriate criteria upon which to judge the results are essential to the evaluation process.

In 1993, Van Dyk et al.^(6)^ reviewed the literature with respect to the commissioning and quality assurance of TPSs and provided recommendations for all aspects of these processes. Task Group 23 of the American Association of Physicists in Medicine (AAPM)^(7)^ took the approach of providing beam data for 2 nominal photon energies (4 MV and 18 MV), together with a set of 13 test cases, complete with measured dose values at selected positions. Algorithm accuracy was to be evaluated by modeling each test situation with the TPS and by comparing the results obtained with measured data.

Several reports using the TG‐23 dataset to evaluate various TPSs can be found in the literature.^(^ ^8^ ^–^ ^11^ ^)^ Unfortunately, as linear accelerators changed, acquiring new capabilities such as independent jaws, multileaf collimators (MLCs), and dynamic (or virtual) wedges, and as various photon energies became common, the original TG‐23 dataset became outdated. In response, new datasets have been produced to address the extended capabilities and varying photon energies.^(^ ^12^ ^,^ ^13^ ^)^

Currently, recommendations are available from several European organizations.^(^ ^14^ ^–^ ^16^ ^)^ Within the framework of the Netherlands Commission on Radiation Dosimetry, Venselaar et al.^(13)^ described a system of acceptability criteria based on regions of high/low dose gradient and high/low dose, further subdivided into simple, complex, or more complex geometries.

On the North American scene, the seminal work is the report on quality assurance for TPSs published by the AAPM Task Group 53,^(17)^ of which algorithm validation forms a relatively small part. Regions of analysis are based on the Van Dyk et al.^(6)^ methodology of separating the buildup, penumbral, and inner and outer beam regions. Acceptability criteria, expressed as a percentage of the central‐axis normalization point, usually at a depth of 10 cm, are suggested for each of the regions and are presented in 10 scenarios. The report also includes a recommendation for the accuracy of absolute dose as reported by the TPS at the prescription point, and a caveat to the effect that the criteria are based on the expectations of the authors and should not be used as goals or requirements. Instead, they recommend that the user determine acceptable criteria specific to the particular implementation and situation contemplated. Nevertheless, the Task Group 53 criteria have become a useful benchmark against which TPS algorithms may be evaluated.

Fogliata et al.^(18)^ recently published a study that highlights the ability of the AAA to reproduce measured beam data required for beam configuration. Their study concluded that, if the clinic‐measured data for a particular photon beam is used to configure the AAA, then the calculated percentage depth doses (PDDs) will be within 1% (beyond $D_{\max}$) or 1 mm (before $D_{\max}$) of the PDDs used for beam configuration.

Van Ecsh et al.^(19)^ recently published the combined acceptance testing report from three cancer clinics in which the AAA algorithm was tested in a wide variety of clinical conditions such as with open and wedged, asymmetric, MLC‐shaped, and intensity modulated beams and with inhomogeneous media. However the analysis techniques and criteria were different from those of TG‐53.

The present work describes a joint effort undertaken by the medical physics departments of two comprehensive cancer clinics to validate the AAA photon‐dose calculation algorithm for a broad range of clinically relevant situations, using as a basis test cases described in detail by Gifford et al.^(20)^ Those tests, the analyses, and the acceptability criteria were in large part based on the report of the AAPM Task Group 53. The outcomes of those tests were also compared to those provided by Gifford et al.^(20)^ for another commercial TPS (Pinnacle^3^, version 4.2: Philips Medical Systems, Andover, MA). For some test situations, additional measurements were included to evaluate the algorithm more thoroughly. Some insight into the suitability of these criteria is also provided here.

It should be noted that, unlike the works of Fogliata et al.^(18)^ and Van Esch et al.,^(19)^ in which the data required for beam configuration were measured and entered into the AAA's configuration module, the present work tested the ability of the AAA configured with “golden beam data” to reproduce measurements on beams matched to standard data. Therefore, a secondary objective of the present work was to provide insight into the variability that might be encountered when measured results obtained from similar models of accelerators produced by the same manufacturer are compared with each other and with the “golden beam data.”

## II. MATERIALS AND METHODS

Workstations loaded with the Eclipse TPS running the AAA photon dose calculation algorithm software version 6.5 (No. 7514, Application build 7.3.10sp3) were delivered to the physics departments of the Tom Baker Cancer Center (TBCC) and the Cross Cancer Institute (CCI) with radiation beams factory configured to match the Varian golden beam data for 6 MV and 15 MV. All measurements were carried out on Varian 21EX accelerators operating with photon energies of 6 MV and 15 MV, matched to Varian golden beam data,^(^ ^21^ ^,^ ^22^ ^)^ and equipped with 120‐leaf Millennium MLCs. The accelerator at the CCI was commissioned at installation to match the golden beam data,^(^ ^21^ ^,^ ^22^ ^)^ but no specific attempt was made to do so at the TBCC. The specification of golden beam data includes $D_{\max}$ (6 MV: 1.6±0.15 cm; 15 MV: 2.9±0.15 cm) and relative dose at 10 cm depth (6 MV: 67.0%±1%, 15 MV: 77.0%±1%) for 10×10‐cm field size as well as beam flatness (±2.5%) and symmetry (2.0%) for 40×40 cm field size. The measured values for the CCI 21EX unit of $D_{\max}$ (6 MV: 1.63 cm; 15 MV: 2.84 cm), relative dose at 10 cm depth (6 MV: 66.9%; 15 MV: 76.8%), flatness (6 MV: 2.5%; 15 MV: 2.2%), and symmetry (6 MV and 15 MV: 1%) indicated that the unit was matched to the golden beam specifications.^(^ ^21^ ^,^ ^22^ ^)^ The TBCC unit was similarly matched to the golden beam specification as indicated by the following measurements: $D_{\max}$ (6 MV: 1.67 cm; 15 MV: 3.06 cm), relative dose at 10 cm depth (6 MV: 67.7%; 15 MV: 77.8%), flatness (6 MV: 2.0%; 15 MV: 2.0%), and symmetry (6 MV and 15 MV: 1%).

Validation measurements fell into one of three categories (Table 1):

- Relative dose comparisons (test cases 1 – 10)
- Absolute dose comparisons (test cases 1 – 10)
- Anthropomorphic phantom measurements (test cases 11 and 12)

The methodology used was largely that described by Gifford et al.^(20)^, with a few modifications where necessary or when descriptions were not sufficiently complete.

**Table 1:** Summary of test cases, all fields with a source‐to‐surface distance (SSD) setup

Test case	SSD (cm)	Gantry angle	Field sizes (degrees)	Beam modifier (cm)
1. Open square fields^a^	100	0	5^2^, 10^2^, 25^2^	None
2. Extended SSD square fields^b^	125	0	8^2^, 20^2^	None
3. Rectangular fields	100	0	5×25, 25×5	None
4a. Wedged square fields^c^	100	0	6^2^, 20^2^, 15^2^	45 degrees, 60 degrees
4b. Open and wedged with 45‐degree collimator	100	0	20^2^	None, 45 degrees
5. Cerrobend‐blocked^d^ mantle field	100	0	30^2^	Block (Fig. 1)
6. Isocentric, 10×10 cm at surface	6 MV: 90 15 MV: 80	0	11.1^2^ 12.5^2^	None
7. Oblique incidence	100	305, 330	10^2^	None
8. Asymmetric half‐beam,^e^ open and wedged	100	0	10:10, 10:0	None, +45 degrees
9. Oblique incidence, 45‐degree wedge	100	315	10^2^	Wedge
10. Multileaf collimator (MLC)	100	0	Triangle (Fig. 2)	MLC
11. CIRS phantom^f^ and pinpoint chamber (CCI)	100	0	26×14	None
12. Rando phantom,^g^ with thermoluminescent dosimeter (TBCC)	100	Thorax: 0 Neck: 270	12×20	None 10×16

a At CCI, 10×10 cm only.

b Field defined at 100 cm.

c Field 15×15 cm with 60‐degree wedge only.

d Cerro Copper and Brass Company, Belfonte, PA.

e Jaw defined half‐beam collimation.

f CIRS, Norfolk, VA.

g The Phantom Laboratory, Salem, NY.

CCI=Cross Cancer Institute; TBCC=Tom Baker Cancer Centre.

### A. Relative dose comparison

**Figure 1:**
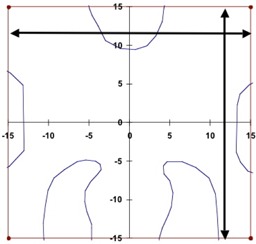
The outline of the mantle field used in test case 5, including the block shape. This field was used to measure the central‐axis inline and crossline profiles at each depth (see text). The off‐axis profiles were measured along the lines with the arrows each at 12 cm distance from the central axis.

**Figure 2:**
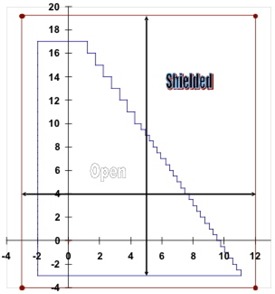
The outline of the multileaf collimator field used in test case 10, including the jaw settings at X1=3 cm, X2=12 cm, Y1=3 cm, and Y2=19 cm. This field was used to measure the central‐axis inline and crossline profiles at each depth (see text). The off‐axis profiles were measured along the lines with the arrows: inline at 5 cm and crossline at 4 cm distances.

Relative dose measurements were made in water using Wellhofer scanning systems (OmniPro‐Accept, versions 6.2 and v6.3: Wellhofer, Schwarzenbruck, Germany) and CC13 (Wellhofer) ion chambers. Figs. 1 and 2 show details of the field outline for the cerrobend‐shaped mantle field in test case 5 and the MLC‐shaped triangular field in test case 10. Off‐axis profiles were measured along the lines marked by arrows.

All scans were taken at 1.2, 4.0, 10.0, 20.0 cm depths for the 6‐MV beam and at 3.2‐, 6.0‐, 10.0‐, 20.0 cm depths for the 15‐MV beam. For each of the test cases, a scan sequence was programmed as follows:

- Crossline (transverse) and in‐line (radial) scans at 4 depths through the beam central axis and repeated at off‐axis planes located at 80% of the distance to the field edge for each depth. Off‐axis crossline scans were closer to the Y2 jaw (toward the gantry), and off‐axis inline scans were closer to the X2 jaw (gantry right, collimator 0 degrees, Varian IEC convention).
- For the asymmetric beam configurations, additional inline scans were programmed at similar off‐axis planes on the opposite side.

Experimental setup and beam energy consistency were checked daily by measuring the PDD of a 10×10‐cm field (SSD=100 cm, 6 MV only).

With the following exceptions, every specific test group (constant setup and beam energy) of profiles was normalized to the central axis point at 10 cm depth, with all stated depths measured perpendicular to the phantom surface. If the beam entry point on the water surface is designated as x=0 (along the 0‐degree gantry crossplane) and y=0 (perpendicular depth), then the oblique incidence test cases were normalized as follows:

- Test case 7 was normalized at x=0 with y=6.0 cm for gantry angle 330 degrees and y=4.0 cm for gantry angle 305 degrees.
- Test case 8, the asymmetric half‐beam, was normalized to a point located at x=5.2 cm off‐axis towards the open jaw at a depth y=10 cm (that is, approximately at the midpoint of the exposed field).
- Test case 9, with a gantry angle of 315 degrees, was normalized at x=5.2 cm and depth y=5 cm.

For open, symmetric beam profiles, the probe centering was checked with the 50% field edges, and no more than 0.3 mm relative shift was found. For these fields, centered profiles were reflected about the central axis and then averaged to reduce the influence of minor asymmetry.

For the comparison of measured and calculated profiles, we chose to calculate the number of points passing the criteria in a manner that would allow for comparison to previously published data. Gifford et al.^(20)^ presented a comparison of measured dose data with calculations provided by an established TPS (Pinnacle^3^, version 4.2). By keeping the measurement setup, field size, beam modifying devices, and evaluation scheme similar to those used by Gifford's group^(20)^, we were able to compare the overall performance of the two TPSs within the framework of the tests summarized in Table 1. To perform this comparative evaluation, we had to divide the measurement space into buildup, outer beam, penumbra, and inner beam regions in a manner similar to that of Gifford et al.^(20)^ and as also given in TG‐53. Table 2 gives the tolerances used in the evaluation. In accordance with TG‐53, tolerances are based on beam configuration and measurement region. In the buildup and inner and outer beam regions, the percentage dose difference was used as a comparison, calculated as

(1) $$\%{\text{Dose}}_{\text{difference}}=\%{\text{Dose}}_{\text{measured}}-\%{\text{Dose}}_{\text{AAA}},$$

where each term is expressed as a percentage of the test case normalization point.

**Table 2:** Tolerances^a^ used in the validation of the analytical anisotropic algorithm

Test case	TG‐53 situations	Inner (%)	Penumbra (mm)	Outer (%)	Buildup (%)	TSF (%)
1. Open square fields	Square fields	1.5	2	2	20	0.5
2. Extended SSD square fields	SSD variations	1.5	2	2	40	1
3. Rectangular fields	Max (rectangular, square fields)	2	2	2	20	0.5
4a. Wedged square fields	Wedged	5	3	5	50	2
4b. Wedged, with 45‐degree collimator	Wedged	5	3	5	50	2
5. Cerrobend‐blocked^b^ mantle field	Blocked	3	2	5	50	1
6. Isocentric, 10×10 cm at surface	Square fields	1.5	2	2	20	0.5
7. Oblique incidence	Max (SSD variations, external surface variations)	3	2	5	40	1
8. Asymmetric half‐beam	Asymmetric	3	2	3	20	1
9. Oblique incidence, 45‐degree wedge	Wedged	5	3	5	50	2
10. Multileaf collimator (MLC)	MLC	3	3	5	20	1

a The value of each tolerance was obtained from $\text{TG}-53^{20}$, published by the American Association of Physicists in Medicine.

b Cerro Copper and Brass Company, Belfonte, PA.

TSF= total scatter factor (defined in section III.B); SSD= source‐to‐surface distance.

Depending on the scan length and sampling rate, the spacing between Wellhofer‐measured data points ranged from 0.1 mm to 0.5 mm. The calculation grids for TPSs are coarser, and in this study, the spacing was 2 mm at the CCI and 2.5 mm at the TBCC. As a result of mismatch between measurement and calculation grid spacing, either the calculated data needed to be interpolated on the measurement grid points (TBCC approach) or the measured data needed to be interpolated on the calculation grid points (CCI approach). As a result of the foregoing differences, the TBCC results contain 5 – 7 times as many comparison points as do the results from the CCI. However, it was still possible to compare results between the two centers based on the percentage of points passing each evaluation criterion.

### B. Absolute dose comparison

To validate the accuracy of MU calculations for the AAA, CC13 ionization chambers were used to measure dose at the normalization point for each test case and, at the same session, for the standard case of a 10×10‐cm field at 10 cm depth with an SSD of 90 cm. The ratio of these values is defined to be the total scatter factors (TSF). Per Gifford et al.,^(20)^ measured and calculated total scatter factors (TSF) were compared rather than the absolute number of MUs used to deliver a specified dose. The rationale behind this approach is that the latter quantity corresponds to the TG 51 calibration point, and its measurement can be taken at the same time as the dose normalization point is measured for each test, thus alleviating errors attributable to day‐to‐day fluctuations in machine output. The percentage difference, ΔTSF, between the measured $\left({\text{TSF}}_{\text{measured}}\right)$ and AAA‐calculated $\left({\text{TSF}}_{\text{AAA}}\right)$ TSFs was computed as

$$\Delta \text{TSF}=100\%\times ({\text{TSF}}_{\text{measured}}-{\text{TSF}}_{\text{AAA}})/{\text{TSF}}_{\text{measured}}.$$

### C. Anthropomorphic phantom measurements

The different anthropomorphic phantoms available at the two centers meant that independent methodologies were used to perform the evaluations of the calculated and measured doses in inhomogeneous media.

**Figure 3:**
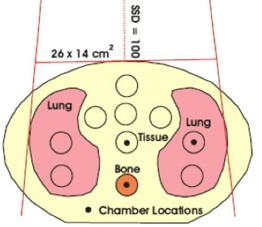
Locations of the test points within the CIRS phantom (CIRS, Norfolk, VA). Source‐to‐surface distance is 100 cm, field size is 26×14 cm, 500 monitor units. The yellow background is the tissue‐equivalent material.

At the CCI, for test case 11, 500 MUs were delivered in a 26×14‐cm anterior field at both 6 MV and 15 MV to the inhomogeneous CIRS IMRT verification phantom (Model 002LFC: CIRS, Norfolk, VA), and ion chamber measurements were made using a pinpoint chamber (N31006: PTW Freiburg, Freiburg, Germany) and an electrometer (Unidose: PTW Freiburg). This particular phantom simulates the human thoracic region, with simulated lung, soft tissue, and cylindrical vertebrae made from a bone analog. Three measurement points, one within each of the three materials, were selected as shown in Fig. 3. Chamber readings were converted to dose by comparison with chamber readings at $D_{\max}$ in solid water for a 10×10‐cm beam and at a distance of 100 cm from the source. The AAA‐calculated dose reported is the average dose within small regions of interest contoured to represent the chamber.

**Figure 4:**
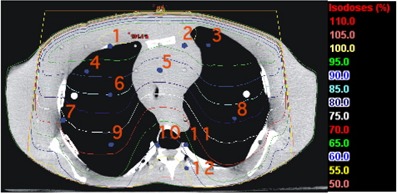
Isodose distribution and location of the test points within the anthropomorphic phantom for the lung test case. SSD=100 cm, 12×28‐cm field, 15 MV, 275 monitor units.

**Figure 5:**
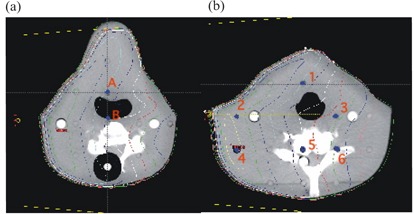
Isodose distributions and location of the test points within the anthropomorphic phantom for the neck test case. (a) Slice 8, 2.5 cm superior to the beam‐entry plane, and (b) slice 9, containing the beam‐entry point. Source‐to‐surface distance is 100 cm, field size is 10×16 cm, 6‐MV beam, 275 monitor units.

At the TBCC, TLD measurements were performed in an Alderson Rando anthropomorphic phantom (The Phantom Laboratory, Salem, NY), which consists of a human skeleton molded into humanoid‐shaped tissue‐equivalent material. Test locations, as indicated by the blue dots in Figs. 4 and 5 were loaded with capsules containing sufficient TL100 powder for three readings, and 275 MUs were delivered for each of two test cases.

In test case 12a, a 15‐MV anterior beam (gantry: 0 degrees; SSD: 100 cm) with a field size of 12×28 cm was set at the center of a thorax section (slice 15) with 12 TLDs inserted in the central‐axis plane.

In test case 12b, a 6‐MV lateral beam (gantry: 270 degrees; SSD: 100 cm) with a field size of 10×16 cm was set at the center of a neck section (slice 9) with 6 TLDs near the central‐axis plane and 2 TLDs 2.5‐cm superior (slice 8).

The calibration TLDs were irradiated at 10 cm depth in both the 6‐MV and 15‐MV beams. Calculations were performed at corresponding points using both the Eclipse AAA and the Pinnacle^3^ CCC algorithm (version 6.2b, Philips Medical Systems). Both TPSs used a CT density conversion table measured specifically for the scanner used (PQ5000, Philips Medical Systems). The % ${\text{Dose}}_{\text{difference}}$ relative to measured dose was calculated as follows:

(2) $$\%{\text{Dose}}_{\text{difference}}=100\%\times ({\text{Dose}}_{\text{measured}}-{\text{Dose}}_{\text{EclipseAAA}\;\text{or}\;\text{Pinnacle}\;6.2b})/{\text{Dose}}_{\text{measured}}.$$

For the inner beam region in heterogeneous media, TG‐53 specifies a tolerance of 7%.

## III. RESULTS AND DISCUSSION

Measurements and calculations using the Eclipse AAA for relative dose profiles, TSFs, and anthropomorphic phantoms are compared and presented separately.

### A. Relative dose comparison

Approximately 700 profiles at 6 MV and 15 MV were measured by each institution for test cases 1 – 10 and evaluated using pass/fail criteria for the various subregions given in Table 2. Table 3 shows the overall percentage of the total number of points passing the criteria in all test cases performed for a given energy at each institute. The overall result is calculated as follows:

$$overall\;results=\frac{\sum_{all\;test\;cases}points\;passing\;test\;case_{i}}{\sum_{all\;test\;cases}points\;in\;test\;case_{i}}\;\;\cdot$$

When individual test cases are combined in this manner to obtain an overall result, the comparative results for the small field sizes are reduced in importance relative to the large field sizes, because large fields contribute more points. The data are presented in this way to permit comparisons with the results provided by Gifford et al.^(20)^

**Table 3:** Results^a^ of the comparisons between measurements and AAA calculations for 6‐MV and 15‐MV beams at the Cross Cancer Institute (CCI) and the Tom Baker Cancer Centre (TBCC)

Center	Energy	Outer (%)	Penumbra (%)	Inner (%)	Buildup (%)
CCI	6 MV	95	85	81	100
	15 MV	97	91	97	100
TBCC	6 MV	97	99	96	99
	15 MV	98	99	98	100

a Overall percentage of points meeting the criteria in test cases 1 – 10 in each region.

Results from the TBCC and the CCI were not combined because of the slight difference in the methods used for comparison. Table 4 provides a summary of all test cases combined for 6‐MV and 15‐MV photon energies at each institution, and the results from similar test cases obtained by Gifford et al. using 6‐MV and 18‐MV photon energies in testing Pinnacle version 4.2.

**Table 4:** Overall results^a^ of the comparison between measurement and analytical anisotropic algorithm calculations

Situations	Outer (%)	Penumbra (%)	Inner (%)	Buildup (%)
CCI 6‐ and 15‐MV combined	96.0	88.0	89.2	99.9
TBCC 6‐ and 15‐MV combined	97	99	97	99.8
Gifford et al.,^(20)^ Pinnacle v4.2	88	93	90	99

a Percentage of points meeting the criteria at each institution [Cross Cancer Institute (CCI), Tom Baker Cancer Centre (TBCC)]. Results from Gifford et al.^(20)^ are presented for comparison.

Tolerances of 20% – 50% in the buildup region (suggested by TG‐53) would appear to be too loose, because our percentage agreement for this region substantially exceeded the agreement for the other regions investigated. The buildup region is a high dose gradient region where distance‐to‐agreement criteria would better suit. We advocate a tolerance of 2 – 3 mm, mirroring the penumbra criteria, with the greater value applicable to wedged and large or asymmetric fields, as suggested by Venselaar.^(13)^

One of the major motivators for undertaking this study was the potential viability of the golden beam data for commissioning the TPSs. One of the largest impacts of a TPS purchase is the need to re‐commission each LINAC within a treatment facility for the new system. It was surprising that the CCI LINAC, which is matched to golden beam data according the specifications provided in the introduction of section II, performed poorly as compared with the TBCC LINAC. That finding suggests that the criteria used for LINAC matching to golden beam data allow for greater variation than do the criteria used in the present analysis, which is based on TG‐53. In general, the analysis of relative dose distributions compare favorably with those reported by Gifford et al.^(20)^ for Pinnacle v4.2. However, a few notable issues are related to penumbra modeling and off‐axis profiles.

#### A.1 The 6 MV off‐axis profiles at 4 cm depth

**Figure 6:**
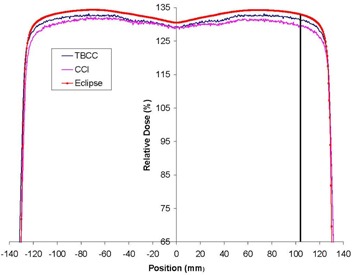
The crossplane profile from golden beam data is shown, together with the measured data from the Tom Baker Cancer Centre (TBCC) and Cross Cancer Institute (CCI) for a 6 MV 25×25‐cm open field at 4 cm depth. The line indicates the location of the off‐axis profile at 80% field edge.

Off‐axis profiles for open beams in or near the buildup region at the CCI revealed systematic disagreements, as compared with Eclipse, greater than the 2% tolerance. The poorest agreement occurred at depths near the buildup region (4 cm for 6‐MV beams and 6 cm for 15‐MV beams). The AAA predicted a larger dose than that measured. A number of the 6‐MV off‐axis profiles at the CCI failed at all inner points because the entire inner portion of the profile was marginally outside the tolerance. Fig. 6 presents the crossline profiles from the golden beam data, TBCC LINAC, and CCI LINAC for a 25×25‐cm field at 4 cm depth. The TBCC profile is closer to the golden beam profile. Fig. 6 also shows the location of the off‐axis inline profile at the 80% field size. Because the point shown by the line in Fig. 6 for the CCI profile deviates from the golden beam profile by more than 2% (the TG‐53 tolerance), an off‐axis inline profile taken at this position resulted in all points within the inner beam regions failing the TG‐53 criteria. The TBCC data are also clearly just inside the 2% tolerance.

These discrepancies resulted in the poorest agreement for the CCI's 6‐MV beams: fewer than 80% of the points in the inner region passed. Notably, poor agreement in the inner beam region of the CCI data occurred not because, on average, 20% of the points in the profiles failed to meet the criteria. Instead, the trend showed either that a profile passed nearly 100% of the points or that it failed nearly 100% of the points.

The flatness and symmetry of the beam were within the TG‐40 requirements^(23)^ (specified to be 3%) and measurements of golden beam specific points (see the introduction to section II) were within specifications. Nevertheless, the inner beam data did not meet TG‐53 requirements at shallow depths. The measurement for the data for test cases 1 and 2 was repeated at the CCI so as to ensure lack of systematic error. The repeated test cases 1 and 2 showed no improvement in the results for the inner beam region. The poor agreement in the inner beam region at the CCI is thus related to the difference in the golden beam and measured profiles.

As a preliminary investigation, test cases 1 and 2 were re‐evaluated after the 6‐MV and 15‐MV beams were reconfigured using the measured input data instead of the “golden beam data.” For test case 1 at 6 MV, the percentage of points passing the criteria in the inner beam region improved from 57%, 66%, and 64% to 68%, 93%, and 100% respectively. Similarly, for test case 2 (6 MV) inner beam data were improved from 63% and 65% to 77% and 99% respectively. These improvements indicate that if Eclipse were to be commissioned with locally measured data, then the results for TG‐53‐based tests would likely improve. The four parameters used for LINAC matching to golden beam data (mentioned in the introduction to section II) seem inadequate for providing a complete match.

#### A.2 Penumbra modeling

**Figure 7:**
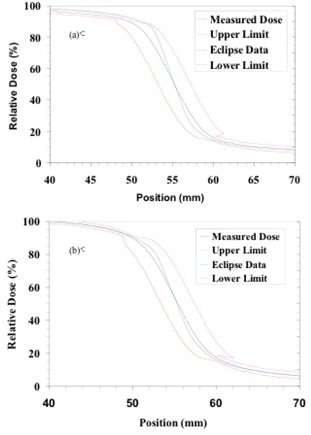
Graphs showing the penumbra portions of measured (blue) and calculated (green) profiles in (a) 6‐MV and (b) 15‐MV beams at 10 cm depth for a 10×10‐cm field. The other two curves in these graphs indicate the lower (red) and upper (magenta) limits according to penumbra criteria given in Table 2. Notice that the Eclipse data show a steeper penumbra than do the measured profiles at both energies. This general trend was observed at the other depths investigated in the present study.

As illustrated in Fig. 7, the AAA‐calculated penumbra in all cases was steeper than the measured penumbra.

**Figure 8:**
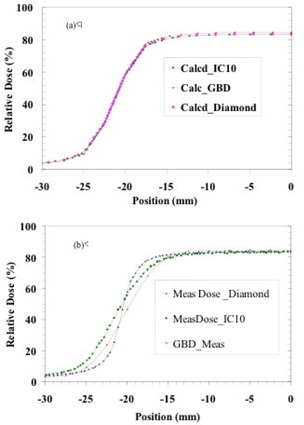
The description of (a) calculated and (b) measured penumbrae of a 4×4‐cm field is shown for three different cases: using a diamond detector; using an CC13 chamber; and data present in the AAA beam configuration denoted as GBD. The calculated data in the upper‐panel curves are obtained from Eclipse after the AAA is configured with the data measured by various detectors. The insensitivity of the modeled penumbra to the measured penumbra is clearly visible in (a). All curves show percentage dose relative to a 10×10‐cm field at maximum dose $\left(D_{\text{max}}\right)$.

A further investigation was carried out at the CCI to assess the influence of detector size on the penumbra modeling. Fig. 8(b) shows the measured penumbra of a 4×4‐cm field for 6 MV with a diamond detector, CC13 chamber, and the measured golden beam data, denoted as GBD. The penumbra differences of the measured data for those detectors are clearly visible with the diamond detector profile showing the steepest penumbra. These measured data were separately used for beam configuration and, for each case, the profile calculated by the AAA was obtained.

Fig. 8(a) shows the profiles. The calculated profiles nearly overlie each other in the penumbra region. The AAA‐calculated penumbra is thus not sensitive to the data used in its configuration. It would appear that the penumbra modeled by the AAA lies somewhat between those measured by a point‐like detector and by a relatively large‐volume ion chamber such as the CC13. Therefore, we expect that our measurements suffered from the usual volume averaging because of the finite inner diameter (6 mm) of the CC13 ion chambers.

As shown by Dawson et al.^(24)^, the 80% – 20% penumbra width measured with a 6‐mm diameter chamber would be 3 mm larger than the width measured with an infinitely narrow detector for 6‐MV photons. This observation is generally supported by the data in Fig. 8(b). The pass rate in the penumbra region was more affected in the CCI data than in the TBCC data because of the fewer number of points available for comparison as described in subsection II.A.

Because the modeled penumbrae of the AAA appear to be insensitive to the measured data, further improvements in penumbra evaluation are not anticipated, and in fact, we expect that the AAA data more closely represent the actual beam penumbra.

### B. Absolute dose comparison

As discussed earlier, TSF was used for absolute dose comparison and is defined as the machine output at the dose normalization point for each test case divided by the output of a 10×10‐cm field at 10 cm depth, and an SSD of 90 cm.

Table 5 summarizes the differences in TSF between measurements and AAA calculations. The agreement achieved is very good. The TSFs as calculated by the AAA are, on average, slightly lower than the measured values, with the average of all results being between 0.2% and 0.4% lower, depending on the institution and energy. For the most part, the only discrepancies greater than 2% are those associated with physical wedges. That finding is to be expected because of possible differences in the physical wedges themselves and because of experimental errors in positioning the measurement probe at the center of the physical wedge. Slight differences in wedge material and fabrication may exist, but the relationships between the results shown below for two energies and the two institutions do not support this rationale for the discrepancies. Moreover, the wedge factors used at the two institutions are within 1% of each other. Experimental error in probe centering is a more likely cause and is possibly exacerbated by differences in the position of the focal spot of the accelerator for the two energies.

**Table 5:** Percentage differences^a^ in the total scatter factor (TSF), by institution and energy, between measured values and those calculated by analytical anisotropic algorithm for each test case

	TBCC		CCI	
Test case	6 MV (%)	15 MV (%)	6 MV (%)	15 MV (%)
1. Open square fields	0.4	1.3	0.3	1.0
	—	—	0.6	0.6
	0.5	0.4	0.6	0.7
2. Extended source‐to‐surface distance	0.6	1.5	0.9	1.1
	0.5	0.5	0.5	0.3
3. Rectangular fields	0.3	0.8	0.6	0.9
	0.4	0.8	0.0	0.6
4a. Wedged fields	1.4	1.0	1.4	−0.6
	1.2	1.0	0.5	−0.5
	1.1	−3.0	0.5	−0.6
4b. Open collimator, 45 degrees	0.3	0.1	—	—
Wedged collimator, 45 degrees	1.1	1.1	1.0	−0.5
5. Mantle field	0.5	−0.8	0.9	1.3
6. Isocentric	0.0	−0.6	0.1	0.2
7. Oblique incidence	−1.4	−0.6	−1.3	0.4
	−1.0	−0.9	−1.0	1.0
8. Asymmetric fields	0.3	−0.2	—	—
	1.4	0.3	−1.0	−2.3
9. Oblique, wedged	−0.7	0.5	0.4	−1.4
10. Multileaf collimator shaped	0.7	1.5	0.7	1.2
Mean	0.4	0.3	0.3	0.2
Range	1.4	1.5	1.4	1.3
	−1.4	−3.0	−1.3	−2.3

a Discrepancies greater than 2% are bolded.

### C. Anthropomorphic phantom measurements

Figs. 4 and 5 show the isodose distributions calculated by the AAA in simulating the irradiation of the Alderson Rando phantom at the TBCC as described in subsection II.C for the thorax and neck sections respectively. Tables 6 and 7 show the results of the TLD measurements. For comparison, Tables 6 and 7 also show the calculated values generated by Pinnacle^3^ 6.2b.

For both sites and both energies, the doses calculated with the Eclipse AAA are slightly higher than the measured dose values. For the thorax section, the mean difference for the AAA is −2.2%, with a maximum discrepancy of −5.2%, and for Pinnacle^3^, the mean difference is −0.2% with a maximum discrepancy of −2.6%. For the neck section, the mean difference for the AAA is −1.6%, with a maximum discrepancy of −5.3%, and for Pinnacle^3^, the mean difference is −0.3%, with a maximum discrepancy of −2.6%.

The algorithms for dose correction (calculation) in inhomogeneous media are completely different between the two TPSs. The Pinnacle dose calculation is based primarily on point‐source dose‐spread array; Eclipse uses pencil beam in association with lateral density scaling.^(^ ^4^ ^,^ ^5^ ^)^ In principle, the point‐spread kernel‐based method allows for greater flexibility in dealing with three‐dimensional (3D) inhomogeneity than do pencil‐beam kernels. In this case, dose at a point from a point source of given TERMA (total energy released per unit mass) at another location in the patient can be calculated by scaling both the primary and the scatter. Point‐to‐point density scaling of this kind is not afforded by the pencil‐beam algorithm. Thus, the Pinnacle system may, in general, be more accurate in inhomogeneous media.

Although these dosimetric tests are certainly challenging, the agreement achieved is reasonable. The slices chosen for the neck section exhibited rapid change in contour, significant obliquity, and missing tissue. Scatter effects were present, as was bone interface. In the thorax section, to challenge the AAA, dosimeters were also placed at lung and bone interfaces. In general, the accuracy of TLD is not better than ±3%.^(25)^ In the present study, the average standard deviation of the three readings was 1.9%. The range was 0.4%−3.5%, with 1 value of 6.3% as a result of loss of TL powder for point 8 of the thorax test.

All differences observed in TLD measurements between the measured and calculated data are within the 7% tolerance recommended by TG‐53.

**Table 6:** Doses measured with a thermoluminescent dosimeter (TLD) in the thorax section of the Rando phantom^a^ as compared with calculations by the Eclipse^b^ analytical anisotropic algorithm (AAA)

		AAA		Pinnacle^3^	
TLD location (Fig. 6)	Measured dose^c^ (Gy)	Calculated dose (Gy)	%Diff^d^	Calculated dose (Gy)	%Diff^d^
1	2.59±2.2	2.67	−3.0	2.63	−1.2
2	2.57±0.8	2.63	−2.3	2.58	−0.4
3	2.62±1.8	2.67	−1.9	2.58	1.8
4	2.40±1.7	2.47	−3.0	2.46	−2.5
5	2.24±0.7	2.29	−2.2	2.28	−1.6
6	2.23±0.4	2.30	−2.8	2.24	−0.3
7	1.98±2.1	2.00	−0.5	1.98	0.2
8	2.10±6.3	2.20	−5.0	2.15	−2.6
9	1.90±1.8	1.96	−2.9	1.89	0.5
10	1.60±2.4	1.58	−1.5	1.57	2.0
11	1.70±1.3	1.70	−0.5	1.67	2.3
12	1.44±2.8	1.52	−5.2	1.45	−0.6
Mean difference			−2.2		−0.2
Maximum difference			−5.2		−2.6

a The Phantom Laboratory, Salem, NY.

b Varian Medical Systems, Palo Alto, CA.

c Standard deviation of three measurements.

d Differences are expressed as a percentage of the measured dose.

**Table 7:** Doses measured with a thermoluminescent dosimeter (TLD) in the neck section of the Alderson Rando phantom^a^ at the Tom Baker Cancer Centre as compared with calculations by the Eclipse^b^ analytical anisotropic algorithm (AAA) and the Pinnacle^3^ treatment planning system,^b^ version 6.2b

		AAA		Pinnacle^3^	
TLD location (Fig. 7)	Measured dose^c^ (Gy)	Calculated dose (Gy)	%Diff^d^	Calculated dose (Gy)	%Diff^d^
A	2.21±3.5	2.21	0.0	2.19	0.9
B	2.03±0.8	2.03	−0.2	2.05	−1.1
1	2.25±1.2	2.22	1.2	2.21	1.7
2	2.57±0.5	2.59	−0.8	2.56	0.5
3	1.68±3.5	1.77	−5.3	1.70	−1.2
4	2.60±2.5	2.65	−1.9	2.62	−0.8
5	1.91±0.4	1.99	−4.2	1.96	−2.6
6	1.64±3.3	1.67	−1.6	1.64	0.4
Mean difference			−1.6		−0.3
Maximum difference			−5.3		−2.6

a The Phantom Laboratory, Salem, NY.

b Varian Medical Systems, Palo Alto, CA.

c Standard deviation of three measurements.

d Differences are expressed as a percentage of the measured dose.

The CCI results for the phantom irradiation are well within the TG‐53‐recommended differences of 7%. Although this test was not exhaustive, the average difference was −1.8%, with the AAA predicting greater dose than was measured (Table 8). The CIRS phantom was also used by Van Esch et al.^(19)^ and irradiated by a lateral beam. Those authors showed that the AAA overestimated the dose by up to 5% for large field sizes, a result that is similar to ours.

**Table 8:** Measured dose and dose calculated by analytical anisotropic algorithm (AAA) in the CIRS phantom^a^ at the Cross Cancer Institute

Energy	Dose point (Fig. 8)	Measurement (Gy)	AAA calculation (Gy)	Difference (%)
6 MV	Tissue	3.29	3.29	0.0
6 MV	Bone	2.43	2.43	0.0
6 MV	Anterior lung	3.86	4.05	−4.8
15 MV	Tissue	3.60	3.73	−3.3
15 MV	Bone	2.90	2.92	−0.5
15 MV	Anterior lung	4.13	4.22	−2.1
—	Mean	—	—	−1.8

a CIRS, Norfolk, VA.

## IV. CONCLUSIONS

The present study used two independent analysis methods to compare dose measurements from two different clinical LINACs with AAA‐calculated doses. As can be seen, the AAA performed well for the conditions tested. Moreover, the results compare well with those published by Gifford et al.^(20)^ for a different TPS.

As compared with results obtained at the TBCC, results obtained at the CCI show that a number of test cases performed poorly in the inner beam and penumbra regions. These differences in the inner beam region possibly result from a mismatch of CCI units to the golden beam data at shallow depths, because the results improved when locally measured data were used for commissioning the TPS. Thus, we conclude that the use of golden beam data may not be adequate to ensure agreement with the tight standards set out in TG‐53 for the inner beam region.

Penumbrae modeled by the AAA are steeper than their counterparts measured with ion chambers, but are also insensitive to this measured input data. True validation in this region should use a very small detector, and the analysis should use a resolution higher than 2 mm.

Seemingly slight differences in analysis technique can affect the results originating in different clinics, as evidenced by differences in the penumbra results seen here, which resulted from the choice of interpolation method.

The exceptionally good results in the buildup region lead to the question of whether the tolerances were sufficiently rigorous. A tighter tolerance using millimeters to agreement may be more appropriate.

The analyses of absolute dose also show good comparisons between the CCI, TBCC, and Eclipse AAA data. Poorest agreement was obtained for physical wedges, which might be the result of differences in the physical wedges at the two institutions and in the “golden beam data.”

Most of the measurements made in one of the two anthropomorphic phantoms that duplicate more clinically realistic conditions were within the tolerances recommended by TG‐53, but these tests were not exhaustive, and our group felt that further measurements would be useful. Further work is also needed to validate additional clinical tools such as enhanced dynamic wedge and IMRT.

## ACKNOWLEDGMENTS

The present work was financially supported by Varian Medical Systems, Palo Alto, California, U.S.A.
